# Dataset of theoretical multinary perovskite oxides

**DOI:** 10.1038/s41597-023-02127-w

**Published:** 2023-04-28

**Authors:** Zachary J. L. Bare, Ryan J. Morelock, Charles B. Musgrave

**Affiliations:** 1 Department of Chemical and Biological Engineering, University of Colorado Boulder, Boulder, CO 80309 USA; 2 Renewable and Sustainable Energy Institute, University of Colorado Boulder, Boulder, CO 80309 USA; 3 Materials Science and Engineering Program, University of Colorado Boulder, Boulder, CO 80309 USA

**Keywords:** Electronic structure, Density functional theory, Scientific data

## Abstract

Perovskite oxides (ternary chemical formula ABO_3_) are a diverse class of materials with applications including heterogeneous catalysis, solid-oxide fuel cells, thermochemical conversion, and oxygen transport membranes. However, their multicomponent (chemical formula ${A}_{x}{A}_{1-x}^{\text{'}}{B}_{y}{B}_{1-y}^{\text{'}}{O}_{3}$) chemical space is underexplored due to the immense number of possible compositions. To expand the number of computed ${A}_{x}{A}_{1-x}^{{\prime} }{B}_{y}{B}_{1-y}^{{\prime} }{O}_{3}$ compounds we report a dataset of 66,516 theoretical multinary oxides, 59,708 of which are perovskites. First, 69,407 ${A}_{0.5}{A}_{0.5}^{{\prime} }{B}_{0.5}{B}_{0.5}^{{\prime} }{O}_{3}$ compositions were generated in the *a*^−^*b*^+^*a*^*−*^ Glazer tilting mode using the computationally-inexpensive Structure Prediction and Diagnostic Software (SPuDS) program. Next, we optimized these structures with density functional theory (DFT) using parameters compatible with the Materials Project (MP) database. Our dataset contains these optimized structures and their formation (*ΔH*_*f*_) and decomposition enthalpies (*ΔH*_*d*_) computed relative to MP tabulated elemental references and competing phases, respectively. This dataset can be mined, used to train machine learning models, and rapidly and systematically expanded by optimizing more SPuDS-generated ${A}_{0.5}{A}_{0.5}^{{\prime} }{B}_{0.5}{B}_{0.5}^{{\prime} }{O}_{3}$ perovskite structures using MP-compatible DFT calculations.

## Background & Summary

Perovskite oxides (ternary chemical formula ABO_3_) are compelling materials due to their functionality^1,2^, tunability^3–5^, and broad range of chemical properties^6,7^. They have been used in solid-oxide fuel cells^8–10^, photocatalysis^11–13^ and to mediate CO_2_-to-CO^14–16^ and steam-to-hydrogen solar thermal conversions^17,18^, and could underlie future industrial production of these renewable fuels. Perovskite oxides are defined by networks of corner-sharing octahedra formed from transition metal or metalloid B-site cations coordinated by six oxygen anions (BO_6_). Many cations (typically alkali, alkaline earth metal, or lanthanide series elements) can occupy the A-site positions between octahedra and are accommodated through coordinated distortions of the octahedral network that are referred to as octahedral tilting. Glazer derived 23 tilting modes — as defined by the phases and magnitudes of the octahedral tilting relative to the unit cell axes — unique to ternary perovskites^19^, 15 of which were determined to be symmetrically distinct^20^. The stabilities, electronic properties, redox properties, etc. are strongly influenced by octahedral tilting.

Perovskite properties can be accurately predicted using plane wave-based quantum mechanical methods, such as density functional theory (DFT) when computed for the appropriate Glazer tilt. In particular, high-throughput (HT) DFT investigations have explored hundreds and even thousands of theoretical perovskite oxides. For example, Castelli *et al*.^21^ modeled 5,400 ABO_3_ compositions in the cubic perovskite symmetry, and Emery and Wolverton^22^ *exhaustively* modeled all possible ABO_3_ compositions of 73 metals and semimetals as perovskites using DFT. More recently, Jacobs *et al*.^23^ and Bare *et al*.^24^ modeled *subsets* of the multinary theoretical perovskite space (multinary chemical formula ${A}_{x}{A}_{1-x}^{{\prime} }{B}_{y}{B}_{1-y}^{{\prime} }{O}_{3}$) using DFT. Previous HT multinary DFT investigations were necessarily curtailed due to the combinatorial explosion of candidates. Whereas Emery and Wolverton fully enumerated all 5,329 theoretical ternary perovskite oxide compositions comprised of 73 elements (73^2^ = 5,329), extension to just the stoichiometrically restricted ${A}_{0.5}{A}_{0.5}^{{\prime} }{B}_{0.5}{B}_{0.5}^{{\prime} }{O}_{3}$ theoretical space results in more than 28 million possible perovskites. Characterization of this complete space using DFT would be prohibitively demanding, even foregoing evaluation of the many possible octahedral tilting modes to identify the DFT ground state structure. The immensity of this materials space motivates the development of tools to reduce the computational expense of DFT calculations in HT investigations of multinary perovskite oxides, and databases to avoid duplication of efforts.

One such tool is the machine-learned tolerance factor, τ^25^, which reliably predicts the probability that a composition forms a stable perovskite and can therefore guide HT investigators through the vast multinary space. τ does not, however, predict perovskite *structures* for DFT optimizations. While techniques such as topotactic structure templating — whereby existing perovskite structures are transmuted with elemental substitutions — can generate theoretical perovskites, significant optimization is required if the initial structures poorly approximate the optimized structures. This is exacerbated in increasingly complex multinary perovskites, where more elements are substituted and optimization expense can therefore compound rapidly in HT investigations. Fortunately, structural templating can be avoided for some multinaries using Structure Prediction and Diagnostic Software (SPuDS), which estimates the lattices, site positions, and octahedral tilting of perovskites *with minimal computational expense* and generally reproduces experimental structures^26^. Optimizing SPuDS predicted structures using DFT is thus not unlike the optimization of experimental crystal structures by DFT to generate the entries of the Materials Project (MP) computational materials database^27^. Therefore, SPuDS structures should provide reasonable initial estimates of structures that lower the expense of DFT geometry optimizations.

In this work, we first generated 69,407 ${A}_{0.5}{A}_{0.5}^{{\prime} }{B}_{0.5}{B}_{0.5}^{{\prime} }{O}_{3}$ perovskites using a custom HT python wrapper to SPuDS. We report the formation enthalpies (*ΔH*_*f*_) and decomposition enthalpies (*ΔH*_*d*_) computed relative to MP-tabulated elemental references and competing phases for the 66,516 structures converged with DFT. We show that SPuDS structures match or are in the same structural family as DFT-optimized structures for 56,716 compositions but differ for 6,808 of the 66,516 converged structures (~10%) that relaxed out of the perovskite phase. Finally, we benchmark our dataset’s DFT energies against the MP database and show that our dataset greatly expands the MP’s multinary oxide compositions. The HT SPuDS python wrapper used to generate structures is available on GitHub (see *Code Availability*) and all structures are available as tabulated entries in the MP with the complete metadata available on MPContribs^28^.

## Methods

### Multinary composition selection

**Fig. 1:**
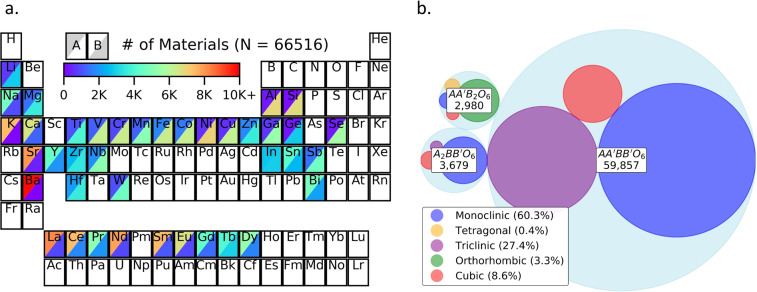
(**a**) Periodic table plot showing the A-site (upper left corner) and B-site (lower right corner) frequencies (heatmap) of each non-radioactive, non-toxic, relatively abundant, and not prohibitively expensive element present in our dataset of 66,516 compositions. (**b**) Breakdown of crystal symmetries following DFT optimization for the three multinary oxide composition types considered: ${A}_{0.5}{A}_{0.5}^{{\prime} }{B}_{0.5}{B}_{0.5}^{{\prime} }{O}_{3}$, $A{B}_{0.5}{B}_{0.5}^{{\prime} }{O}_{3}$, and ${A}_{0.5}{A}_{0.5}^{{\prime} }B{O}_{3}$. Larger circles indicate more frequently observed crystal symmetries.

We identified 69,407 multinary perovskite oxide compositions for DFT optimization using the method described by Bartel *et al*., which is briefly summarized here. To make ${A}_{0.5}{A}_{0.5}^{{\prime} }{B}_{0.5}{B}_{0.5}^{{\prime} }{O}_{3}$ compositions, four unique cations were selected from 39 non-radioactive, non-toxic, relatively abundant, and not prohibitively expensive elements (Fig. 1). For $A{B}_{0.5}{B}_{0.5}^{{\prime} }{O}_{3}$ and ${A}_{0.5}{A}_{0.5}^{{\prime} }B{O}_{3}$ compositions, three unique cations were selected. If the chosen cations had *at least one* combination of common oxidation states that charge-balanced a multinary composition, its tolerance factor τ was computed using Eq. 1. If these cations had more than one combination of charge-balanced, common oxidation states, oxidation states for τ were assigned based on relative cation electronegativities. We only considered cation oxidation states with tabulated bond valence method (BVM) parameters^29^, which SPuDS needs to generate structures.

1 $${\rm{\tau }}=\frac{{r}_{X}}{{r}_{{B}^{\ast }}}-{n}_{{A}^{\ast }}\left({n}_{{A}^{\ast }}-\frac{{r}_{{A}^{\ast }}/{r}_{{B}^{\ast }}}{ln({r}_{{A}^{\ast }}/{r}_{{B}^{\ast }})}\right)$$

In Eq. 1., *r*_A*_ and *r*_B*_ are the stoichiometrically-averaged Shannon ionic radii^30^ of the A/A’- and B/B’-site cations, *r*_*X*_ is the Shannon ionic radius of the X-site cation (oxygen), and *n*_A*_ is the stoichiometrically-averaged A/A’-site oxidation state. For each composition, the cation(s) with the largest ionic radii were assigned to the perovskite A- and/or A’-sites, as larger cations tend to occupy the larger A-sites over the smaller B-sites. The remaining cation(s) were assigned to the perovskite B- and B’-sites. 12-fold, 6-fold and 6-fold coordinated Shannon ionic radii were preferentially chosen for the A/A’-, B/B’- and X-site radii, respectively, to approximate the cubic perovskite structure’s site coordination numbers. In the event that these Shannon radii were not tabulated, τ was computed using the closest available radii. As all compositions considered are within or near the predicted stable perovskite region of τ < 4.18, we generated 69,407 total perovskite structures for DFT optimization.

### Structure generation and DFT optimization

Perovskite structures were generated using PySPuDS, a custom high-throughput python wrapper to the SPuDS program. SPuDS minimizes the BVM Global Instability Index (*GII*) to predict perovskite B-O bond lengths and BO_6_ tilting magnitudes. Morelock *et al*. showed that *GII* correlates with the DFT energies of competing Glazer tilts for ternary ABO_3_ perovskites^31^, meaning that SPuDS computed ternary perovskite oxide structures lower the optimization expense when used as starting structures for DFT optimization. SPuDS constrains structures with multiple B-sites, i.e., $A{B}_{0.5}{B}_{0.5}^{{\prime} }{O}_{3}$ and ${A}_{0.5}{A}_{0.5}^{{\prime} }{B}_{0.5}{B}_{0.5}^{{\prime} }{O}_{3}$ compositions, to have B-site rock salt orderings, but it does not restrict A-site occupancies when multiple A-sites are present in ${A}_{0.5}{A}_{0.5}^{{\prime} }B{O}_{3}$ and ${A}_{0.5}{A}_{0.5}^{{\prime} }{B}_{0.5}{B}_{0.5}^{{\prime} }{O}_{3}$ compositions. Structures with multiple A-sites were therefore given site orderings that minimized the A-site Ewald sums. We attempted to generate all 69,407 structures in the *a*^−^*b*^+^*a*^*−*^ Glazer tilting mode, a decision we justify in the *Comparison to the Materials Project database* sub-section of the *Technical Validation* section. SPuDS does not predict octahedral tilting for 6,523 compositions, meaning these structures were generated in the cubic *a*^0^*a*^0^*a*^0^ Glazer mode. Using the MP-compatible framework detailed in *Computational Methodology*, we converged structures for 66,516 of these 69,407 compositions, which we report here. The remaining 2,891 structures (~4.2% of all compositions considered) had unphysical (exploded) unit cells and/or unreasonable energetics, even after multiple re-optimization attempts, and are not reported in this work.

### Computational methodology

GGA + U DFT calculations were performed using the Vienna Ab-initio Simulation Program (VASP 5.4.1)^32–34^ with the Perdew-Burke-Ernzerhof (PBE)^35^ exchange-correlation functional and projector augmented wave (PAW) pseudopotentials^36,37^ under periodic boundary conditions. All calculations are compatible with the MP database, which tabulates the structures, energetics, and other properties of inorganic materials computed at the PBE (GGA and GGA + U) level of theory. The electronic wave functions were expanded in a plane wave basis set with an energy cutoff of 520 eV. The Brillouin zones were sampled during geometry optimization using the Monkhorst-Pack algorithm^38^ to automatically generate a Γ-point centered k-point mesh with a grid density of at least 1000/(atoms/unit cell). We used pseudopotentials, Hubbard U parameters^39^, magnetic moments, and energy convergence criteria that are consistent with pymatgen’s^40^ MPRelaxSet, which is the default relaxation parameter set for the MP. Consistent with MP’s convergence methodology, we performed two consecutive spin-polarized geometry optimizations, the first of which was initialized in the high-spin ferromagnetic configuration. These geometry optimizations relaxed both the unit cell lattice vectors and atomic positions. Δ*H*_*f*_ and Δ*H*_*d*_ include MP2020 anion corrections that are based on the structure type (i.e., oxide, peroxide, superoxide, or ozonide), as well as element-specific corrections for compatibility between GGA and GGA + U calculations.

### Data scope

Currently, the MP database contains only 3,904 entries composed of the 39 elements considered herein with the chemical formulae ${A}_{0.5}{A}_{0.5}^{{\prime} }{B}_{0.5}{B}_{0.5}^{{\prime} }{O}_{3}$, $A{B}_{0.5}{B}_{0.5}^{{\prime} }{O}_{3}$, or ${A}_{0.5}{A}_{0.5}^{{\prime} }B{O}_{3}$. We report the structures and energetics of 66,516 multinary oxides, which expands this compositional space by well over ten-fold. We anticipate that this dataset will facilitate materials discovery in a space with many current and potential applications, but where DFT calculations were previously sparse. Additionally, and in contrast to other high-throughput perovskite studies that have used template structures or assumed the un-tilted cubic aristotype, we explicitly consider octahedral tilting before DFT optimization. Our investigation into ternary perovskite oxides showed that octahedral tilting typically stabilizes the cubic perovskite phase by more than 100 meV/atom, and by as much as several hundred meV/atom^31^. Thus, explicit consideration of BO_6_ tilting should better approximate ground state perovskite energetics, and more reliably predict perovskite phase stabilities and synthesizability. Finally, we include non-perovskite structures in the dataset and report each structure’s perovskite/non-perovskite classification in the metadata. This is intended to direct materials scientists towards compositions where the perovskite phase is preserved by DFT optimization so that data scientists can use this designation for machine learning. To our knowledge, high-throughput DFT investigations typically do not confirm nor repudiate phase preservation in their metadata, and this has certainly not been previously reported for a multinary perovskite oxide dataset of this size.

### Data properties

**Fig. 2:**
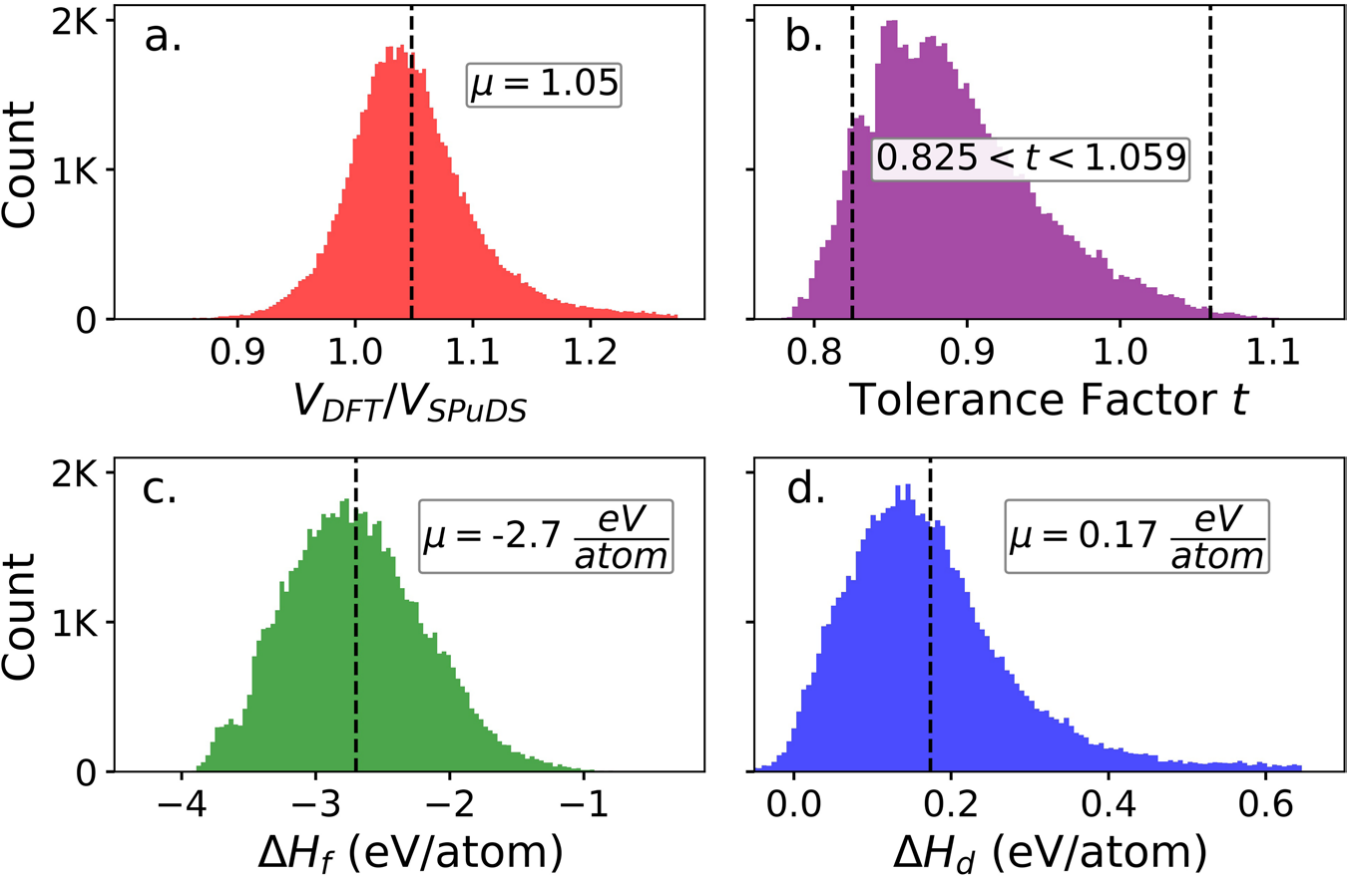
(**a**) Distribution of the ratios of DFT-optimized structure volumes to SPuDS perovskite volumes for the 66,516 compositions in the dataset. (**b**) Distribution of Goldschmidt tolerance factors *t* for compositions in the dataset, computed using Eq. 1. (**c**) Distribution of formation enthalpies (*ΔH*_*f*_) for compositions in the dataset, computed relative to MP tabulated elemental references. (**d**) Distribution of decomposition enthalpies (*ΔH*_*d*_) for the dataset, computed relative to MP tabulated competing phases.

Figure 2a shows the distribution of the ratios of DFT-optimized structure volumes to SPuDS volumes for compositions in our dataset. The mean ratio is 1.05, meaning that SPuDS slightly underpredicts volumes but generally captures multinary perovskite oxide unit cell sizes relative to DFT. Because the SPuDS and DFT volumes are so similar, one might assume that SPuDS structures also accurately estimate multinary perovskite oxide *site positions* relative to DFT. This postulate is evaluated in the *Comparison of SPuDS and DFT-optimized structures* sub-section of the *Technical Validation* section, where we show that site positions predicted by SPuDS approximate DFT optimized site positions for ~85% of compositions in our dataset. The Goldschmidt tolerance factor^41^ predicts experimental perovskite synthesizability for ternary (ABX_3_) compositions from their cation and anion radii. We computed Goldschmidt tolerance factors for *multinary* compositions in our dataset by stoichiometrically averaging their A- and B-site Shannon ionic radii:

2 $$t=\frac{({r}_{A}+{r}_{A{\prime} })/2+{r}_{O}}{\sqrt{2}[({r}_{B}+{r}_{B{\prime} })/2+{r}_{O}]}$$

As shown in Fig. 2b, ~91% of compositions in the dataset are within the 0.825 < *t* < 1.059 reported range of Goldschmidt tolerance factors *t* expected to form perovskites^25^. This suggests that the perovskite phase might not be preferred for ~9% of compositions in our dataset, which we elaborate upon in the *Perovskite or non-Perovskite classification* sub-section of the *Technical Validation* section. Finally, Fig. 2c shows that all 66,516 compositions in the dataset have negative *ΔH*_*f*_ and are thus stable relative to their elemental reference states. Figure 2d shows that for all compositions in the dataset, *ΔH*_*d*_ < 650 meV/atom above the convex hull defined by competing phases in the MP, with a mean *ΔH*_*d*_ of 170 meV/atom. In the *Comparison to the Materials Project database* sub-section of the *Technical Validation*, we indicate when our *ΔH*_*f*_ and *ΔH*_*d*_ values most reliably estimate DFT energetics and justify choosing the *a*^−^*b*^+^*a*^*−*^ Glazer tilt as a surrogate for the ground state structure in HT, theoretical multinary perovskite oxide investigations.

## Data Records

All metadata is hosted by MPContribs (https://contribs.materialsproject.org/projects/Multinary_Oxides^42^) and can be accessed using the MPContribs API and/or downloaded in JSON or CSV formats directly from the host webpage. Each of the 66,516 data entries have a unique *identifier*, which is the composition ordered by A, A’, B and B’ sites, with ‘_’ appended to single-letter element abbreviations (e.g., the unique identifier for A = Tb, A’ = Y, B = Fe and B’ = Co is “TbY_FeCoO6.”) Each entry also has an associated *id* for querying its pymatgen Structure object and metadata from the MPContribs API (e.g., the unique id for “TbY_FeCoO6” is “6346f814660f1387211b46f5”), and a tabulated *formula* for each entry, e.g., TbYFeCoO6. The four sub-headers: *elements*, *oxidation*, *tolerance* and *dH* are placed under the *data* header for each entry. The *elements* sub-header includes the columns *A1*, *A2*, *B1* and *B2*, which tabulate the A, A’, B and B’ cation element identities, e.g., *A1* = “Tb”, *A2* = “Y”, *B1* = “Fe” and *B2* = “Co”. The oxidation sub-header includes the columns *A1*, *A2*, *B1* and *B2*, which tabulate the numerical, positive oxidation states of each cation used to generate SPuDS structures for DFT optimization, e.g., *A1* = 4, *A2* = 3, *B1* = 2 and *B2* = 3 for “TbY_FeCoO6.” The tolerance sub-header includes the columns *t* and *tau*, which are the Goldschmidt^41^ and Bartel^25^ perovskite tolerance factors, respectively (e.g., *t* = 0.802457 and *tau* = 4.19669 for “TbY_FeCoO6”) computed using Eq. 2 and Eq. 1 in this work. Finally, *dH* includes the columns *formation [eV]* and *decomposition [eV]*, which tabulate the formation and decomposition enthalpies (in electron-volts, eV) of the DFT-optimized structures computed relative to only elemental and all competing phases in the Materials Project database, respectively (e.g., *formation [eV]* = −2.70351 and *decomposition [eV]* = 0.0441468 for “TbY_FeCoO6.”) The *perovskite* column is under the *data* sub-header, which specifies “Yes” or “No” if the structure is a perovskite following DFT optimization according to the classification method outlined in the *Perovskite or non-perovskite* *classification* sub-section of the *Technical Validation* section (“Yes” for “TbY_FeCoO6.”) The SPuDS structures optimized with DFT for each entry are available for download as Crystallographic Information Framework (.cif) files under the structures sub-header. All entries in our dataset also have unique “mp-id” identifiers for the Materials Project database, meaning that their DFT-optimized structures and calculation details can be found in the main MP repository.

## Technical Validation

### Comparison of SPuDS and DFT-optimized structures

SPuDS accurately predicts perovskite oxide structures with multiple octahedral cations relative to experimental structures^43^, which we expected would limit the computational expense of our HT DFT investigation. However, SPuDS has not previously been evaluated for a dataset of this scope or scale, i.e., with tens of thousands of DFT-optimized theoretical perovskite oxides with multiple A- and B-sites. We benchmarked SPuDS for HT DFT screening by comparing the structural fingerprint distances (SFPD) and structural similarity metrics (ε) of SPuDS and DFT-optimized structures, as more similar structures should result in lower DFT optimization expense. As implemented in the Matminer python package^44^, a structural fingerprint is a vector of statistical information, i.e., minimum, maximum, mean, and standard deviation, derived from a structure’s coordination environment distributions. The SFPD is the normed difference between two structural fingerprints and quantifies two structures’ similarity; according to the MP, structures with 0 ≤ SFPD ≤ 0.9 are typically similar, whereas structures with SFPD > 0.9 are typically different. As implemented by the AFLOW^45^ software framework’s Xtal-Finder^46^, ε is a non-negative, scalar metric that measures the structural similarity between two structures by computing deviations between crystal lattices (ε_latt_) and mapped site positions (ε_coord_). It also includes a failure term (ε_fail_) for incompatible structures. Hicks *et al*. reported that structures with 0 ≤ ε ≤ 0.1 match and structures with 0.1 < ε ≤ 0.2 reside in the same family, whereas structures with ε > 0.2 are different. Because SFPD and ε are interpretable, scalar quantities, but are derived independently from one another, we compared SPuDS structures to DFT-optimized structures using both metrics.

**Fig. 3:**
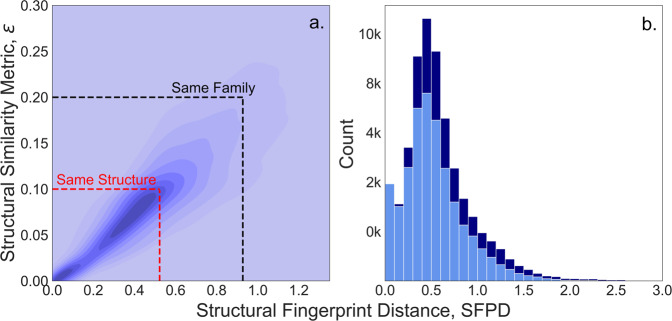
(**a**) Kernel density plot of SFPD vs. ε for the 48,742 compositions in our dataset where ε is computed for SPuDS and DFT-optimized structures. Darker purple coloring indicates higher density regions. By minimizing Gini impurities, we find that ε indicating the same structure (0 ≤ ε ≤ 0.1) corresponds with 0 ≤ SFPD ≤ 0.522 in our dataset, while ε indicating the same structural family (0.1 < ε ≤ 0.2) corresponds with 0.522 < SFPD ≤ 0.927. (**b**) Stacked histogram of SFPDs between SPuDS and DFT-optimized structures for the 66,516 compositions in the dataset (bin width of 0.1). Navy blue indicates compositions that cannot be mapped by Xtal-Finder, whereas light blue indicates compositions that have both a computed SFPD and ε.

Figure 3a plots SFPD vs. ε for the 48,742 compositions in the dataset where Xtal-Finder reports an ε. There is considerable agreement between SFPD and ε; by minimizing Gini impurities using the scikit-learn python package^47^, we find that 0 ≤ SFPD ≤ 0.522 generally corresponds with 0 ≤ ε ≤ 0.1 and 0.522 < SFPD ≤ 0.927 generally corresponds with 0.1 < ε ≤ 0.2, which supports the MP claim regarding SFPD. 25,349 of the 29,969 compositions with 0 ≤ SFPD ≤ 0.522 have 0 ≤ ε ≤ 0.1 (~85%), while 42,305 of the 45,250 compositions with 0 ≤ SFPD ≤ 0.927 have 0.1 < ε ≤ 0.2 (~93%). Xtal-Finder did not map DFT-optimized structures to their starting SPuDS structures for the remaining 17,774 compositions. This does not necessarily mean that the structures for these compositions are dissimilar, however, as 13,549 of the 17,774 unmapped structures have SFPD ≤ 0.927 (Fig. 3b). Rather, this is a limitation of the Xtal-Finder algorithm, which cannot always map the reference structure to the structure to be compared. Based on the general agreement between SFPD and ε, and because SFPD ≤ 0.927 for 56,716 compositions in our dataset (~85%), we conclude that the majority of SPuDS structures either match or are in the same structural family as their DFT-optimized counterparts, meaning that SPuDS reliably estimates multinary perovskite oxide structures for HT DFT. We, therefore, expect SPuDS to, on average, estimate initial perovskite oxide structures that are *at least* within the same structural family as their DFT-optimized counterparts. This should limit the computational expense associated with optimizing perovskite structures for the millions of possible multinary oxide compositions not reported in our dataset.

### Perovskite or non-perovskite classification

The standard MP relaxation set, MPRelaxSet, used for our high-throughput investigation optimizes all internal degrees of freedom (i.e., atomic positions, cell shape, and cell volume) with DFT, which can relax the input SPuDS structure out of the perovskite phase. We classified structures as perovskite or non-perovskite following DFT optimization using the guidelines proposed by Akkerman and Manna for 3D perovskites^48^. These authors define a perovskite to be a “network of corner-sharing BX_6_ octahedra that crystallize with a general ABX_3_ (or equivalent) stoichiometry”, although “deviations from this ABX_3_ stoichiometry can be obtained when the A and B cation sites… are replaced by a combination of other cations” such as in multinary ${A}_{0.5}{A}_{0.5}^{{\prime} }{B}_{0.5}{B}_{0.5}^{{\prime} }{O}_{3}$ compositions.

To check if octahedral connectivity was preserved, we constructed B-site cation octahedral (or non-octahedral) coordination environments from anion nearest neighbors identified using pymatgen’s CrystalNN class^49^. We assigned oxidation states for CrystalNN using the algorithm reported by Bartel *et al*.^25^, meaning that the oxidation states assigned to DFT-optimized structures were the same oxidation states used to generate initial SPuDS structures. This assumes that Bartel’s assignment algorithm is accurate, even though it does not account for charge disproportionation, e.g., mixed valence Bi in Ba(Bi^3+^/Bi^5+^)O_3_^50^, that can be present in perovskite oxide systems. Even when cations with multiple valences are not present, assigning formal oxidation states to DFT-optimized multinary metal oxides is nontrivial and beyond the scope of this work. As a result, perovskite/non-perovskite classifications in our dataset can change depending upon the cation and anion oxidation states assigned, as well as the nearest neighbor finder^51^ and/or the cutoff radius used, although benchmarking suggests that perovskite/non-perovskite classifications only change for a small subset (<1%) of highly sensitive structures.

All 66,516 SPuDS structures input to VASP were deemed perovskite according to our classification methodology. However, only 59,708 DFT-optimized structures were classified as perovskite, whereas the remaining 6,808 structures were classified as non-perovskite (~10%). All 39 elements considered are present in both perovskite and non-perovskite structures. The frequencies of each element’s A- and B-site occupations in perovskites and non-perovskites are tabulated in Supplementary Table 1, where they are organized by the oxidation states assigned by τ that were used to generate their SPuDS structures. Supplementary Table 1 only counts elements once per composition, even if said element occupies both A- and A’-sites ($A{B}_{0.5}{B}_{0.5}^{{\prime} }{O}_{3}$) or B- and B’-sites (${A}_{0.5}{A}_{0.5}^{{\prime} }B{O}_{3}$).

**Fig. 4:**
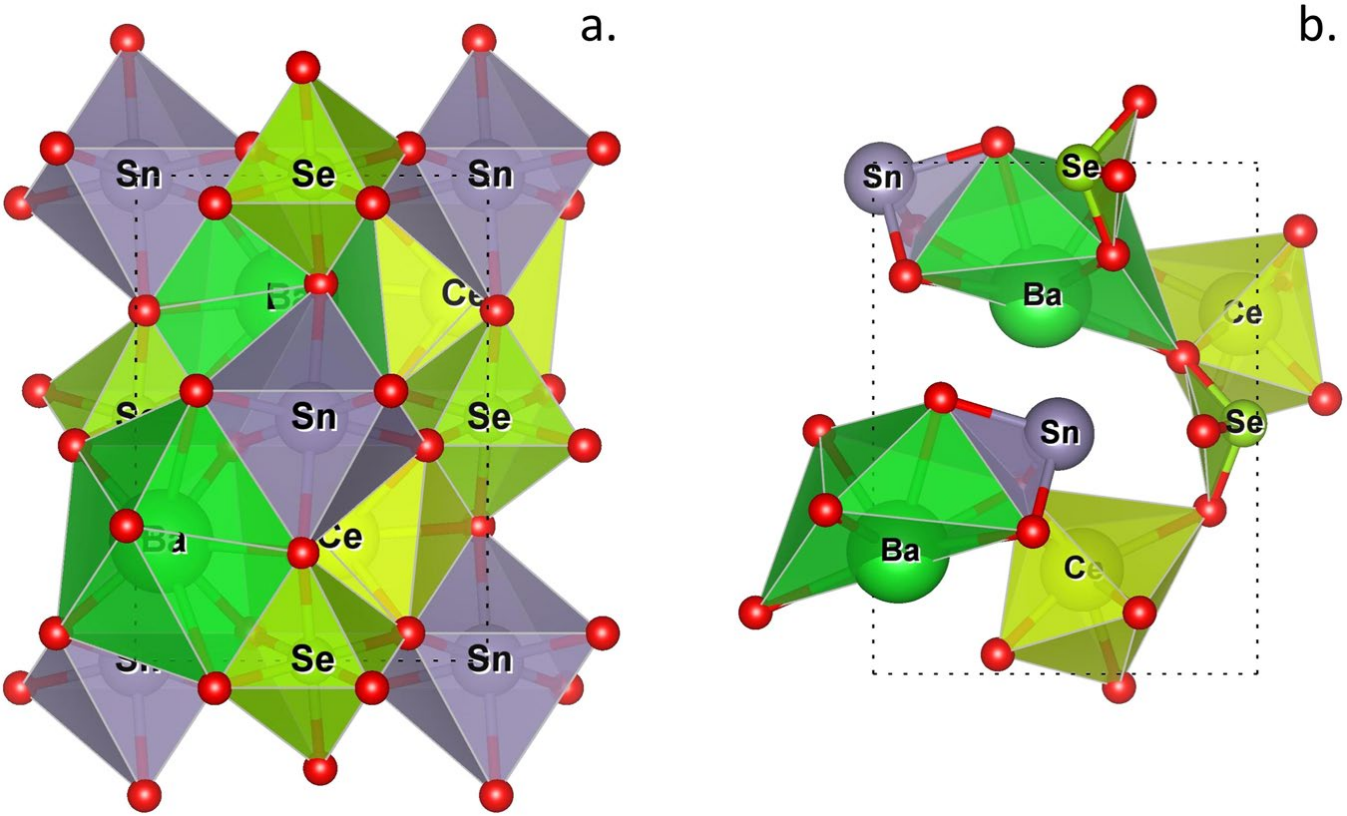
(**a**) The perovskite structure generated by SPuDS in the *a*^−^*b*^+^*a*^*−*^ Glazer tilt for the theoretical perovskite BaCeSnSeO_6_. (**b**) The non-perovskite, DFT-optimized structure of BaCeSnSeO_6_, which no longer has an interconnected network of SeO_6_ and SnO_6_ octahedra following DFT optimization.

DFT optimization can cause under- or over-coordinated B-sites and/or disruption of octahedral connectivities such that not all oxygens remain shared between octahedra, resulting in non-perovskite classifications. For instance, this occurs for the BaCeSnSeO_6_ composition (visualized in Fig. 4. using the Vesta^52^ software package) with Ba^2+^, Ce^4+^, Sn^2+^, and Se^4+^ cations, in which SeO_6_ and SnO_6_ octahedra optimize to SeO_3_ and SnO_3_ complexes. Supplementary Table 1 shows that 1,956 compositions with B-sites occupied by Se^4+^ have non-perovskite DFT optimized structures, while only 1,770 compositions have perovskite structures. This suggests that many Se-containing multinary oxide compositions might preferentially form non-perovskite structures and is consistent with the oxide coordination environment analysis of Waroquiers *et al*.^53^, which determined that Se^4+^ prefers the trigonal non-coplanar environments present in DFT-optimized BaCeSnSeO_6_.

We also used the Robocrystallographer python package^54^, which auto-generates text-based descriptions of crystal structures analogous to a “real-life crystallographer”, to classify structures as perovskite or non-perovskite. Robocrystallographer classified 26,781 of the 66,516 DFT-optimized structures in our dataset as perovskite and 5,907 structures as non-perovskite, with non-perovskite mineral type classifications that include ilmenite (4,065 structures), PbZr_0.5_Ti_0.48_O_3_ (1,201), and hausmannite (255). However, 3,940 of the 4,065 ilmenite structures, 492 of the 1,201 PbZr_0.5_Ti_0.48_O_3_-type structures, and all 255 hausmannite structures are perovskites according to their octahedral network connectivities. Some of these structures have large BO_6_ tilting angles that reduce the A-site coordination numbers to less than eight, which Robocrystallographer interpreted to be “non-perovskite”-like. It therefore returned a non-perovskite mineral type classification, such as ilmenite, *even if* the octahedral network connectivity was preserved in the structure. Another consequence of Robocrystallographer’s mineral type assignment algorithm is that it did not classify 33,828 structures, which is greater than half of our dataset. For this reason, we report perovskite classifications using only the octahedral connectivity rules, and we conclude that ~90% of the converged structures in our dataset are perovskites.

The proportion of compositions in our dataset with structures that are perovskites following DFT optimization generally agrees with the Goldschmidt tolerance factor, *t*, which predicts that ~91% should form perovskites. However, *t* and DFT do not necessarily agree for all compositions in our dataset. For the 59,708 compositions with structures that remain perovskites following DFT, *t* predicts “perovskite” for 54,918 compositions (~92%) and “non-perovskite” for 4,790 compositions. Agreement between *t* and DFT is significantly worse for the 6,808 compositions that relax out of the perovskite structure following DFT, as *t* predicts “non-perovskite” for only 1,155 compositions (~17%) and “perovskite” for 5,653 compositions. As previously discussed, improperly assigned oxidation states---resulting in Goldschmidt tolerance factors calculated from the wrong Shannon radii---could contribute to this observed disagreement. Additionally, *t* does not account for variations in bonding strength or the preferred coordination environments of different elements, and stoichiometrically averaged A/A’ and B/B’ radii might not fully capture the effects of mixed cation sites on octahedral network formation. We also did not optimize non-perovskite starting structures in this work, meaning that some structures that remain perovskite following DFT optimization could have more stable non-perovskite structures that were missed. This is even more likely for cubic *a*^0^*a*^0^*a*^0^ structures where the SPuDS-generated unit cell symmetries limit the atomic degrees of freedom during HT optimization (VASP tag ISYM = 1). Optimizing supercells with more degrees of freedom could improve the poor agreement between *t* and DFT for non-perovskite predictions, albeit by incurring a far greater computational expense.

### Comparison to the Materials Project database

We report *ΔH*_*f*_ and *ΔH*_*d*_ computed relative to MP elemental references and competing phases, respectively, which assumes that the DFT-computed energetics are consistent with the MP and can thus reliably predict perovskite phase stabilities. We quantified the dataset’s MP compatibility by comparing our DFT energetics to MP energetics for structures present in both datasets. There are very few compositions present in the MP repeated in our dataset, let alone repeated *structures*: of the 66,516 multinary oxide compositions reported, only 425 (<1%) have any structural entries, perovskite or non-perovskite, in the MP. Of these 425 overlapping compositions, the MP tabulates at least one perovskite structure for 391 compositions according to the classification rules described in *Comparison of SPuDS and DFT-optimized structures*. We compared energetics for 213 of these 391 compositions, or for the compositions where our perovskite structure matches an MP perovskite structure according to pymatgen’s StructureMatcher class. Because the MP uses spin-polarized calculations, which means that spin magnitudes and orientations are simultaneously optimized with atomic positions, energetics can differ depending upon site spin orderings. For example, Horton *et al*. showed that magnetic sampling with different starting spin initializations was necessary to correctly capture non-ferromagnetic spin orderings for a benchmarking set of 64 materials, 50 of which are mixed metal oxides^55^. Likewise, Bare *et al*. magnetically sampled Gd-containing multinary oxides in their HT DFT investigation because, on average, magnetic sampling stabilized these compositions by ~60 meV/atom relative to initial ferromagnetic ordering^24^. We therefore separately compared our structures that matched or did not match site magnetic moments according to pymatgen’s CollinearMagneticStructureAnalyzer class.

For the 104 compositions in our dataset with structures and magnetisms that matched the MP, the RMSD between DFT and MP energetics is 24.3 meV/atom, which approaches one standard deviation of GGA + U DFT error for oxide formation enthalpies^56^. However, when matching structures with *different magnetisms* were compared, the RMSD increased to 95.3 meV/atom. This nearly four-fold increase confirms that the DFT energetics of our multinary oxides are sensitive to magnetic ordering, most likely because these compositions can have multiple magnetic elements with many unique spin magnitudes and orientations. Due to the unprecedented size of our dataset---where we report more than 80 times as many optimized bulk structures compared to our previous investigation of Gd-containing perovskite oxide redox mediators for solar thermochemical hydrogen production^24^---we forewent magnetic sampling during the first structure optimization and instead restricted all initial spin configurations to be high-spin ferromagnetic to reduce computational expense. The magnetisms for the second structural optimizations were initialized from the final magnetisms of the first structure optimizations and thus were not spin sampled. Because the RMSD attributable to slight differences in final structures, which could arise from spin initialization during structure optimization, is 24.3 meV/atom, or within DFT error (~30 meV/atom), we conclude that high-spin ferromagnetic initializations should be sufficient for structure optimizations. However, high-spin ferromagnetic initializations do not necessarily capture the most accurate DFT total energetics for magnetic structures. Therefore, we recommend single-point magnetic sampling of our converged structures to maximize compatibility with the MP database, with the caveat that sensitivities to magnetic ordering will vary based on the magnetic elements and the number of magnetic sites present in each structure.

**Fig. 5:**
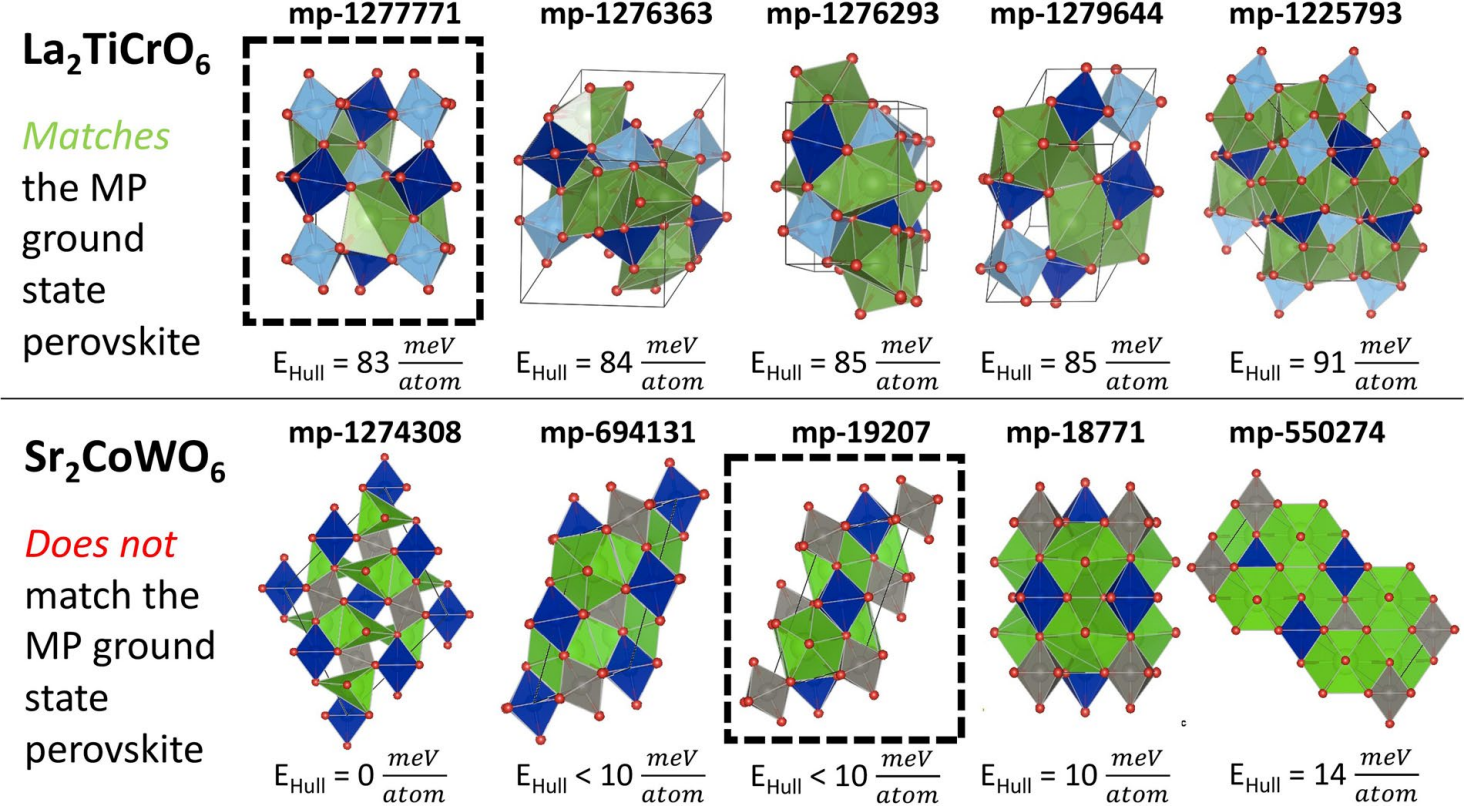
DFT-optimized *a*^−^*b*^+^*a*^*−*^ Glazer tilt structures matches the MP ground state perovskite structure for the La_2_TiCrO_6_ composition but not the Sr_2_CoWO_6_ composition. Both compositions have five unique perovskite entries tabulated in the MP. For 43 of the 52 compositions in our dataset (~83%) with more than one perovskite entry in the Materials Project, our DFT-optimized SPuDS structure matches the MP ground state perovskite structure.

We also benchmarked our choice to constrain all starting structures for DFT optimization to be either the SPuDS *a*^−^*b*^+^*a*^*−*^ Glazer tilt or the SPuDS *a*^0^*a*^0^*a*^0^ Glazer tilt if no tilting was predicted for the *a*^−^*b*^+^*a*^*−*^ Glazer mode. The SPuDS program already estimates Glazer tilt stability using the BVM Global Instability Index; however, because the *GII* does not consider unrealistically short anion-anion (O-O) distances, the *a*^*−*^*a*^*−*^*a*^−^ Glazer tilt is always the lowest *GII* structure, even though the *a*^−^*b*^+^*a*^*−*^ Glazer tilt is more often observed experimentally^57^. Furthermore, our investigation into experimental ABO_3_ perovskite compositions showed that the *a*^−^*b*^+^*a*^*−*^ Glazer tilt optimized to the DFT ground state perovskite for more than 95% of ternaries^31^, and we expected this would also apply to multinaries. To evaluate our choice, we counted the number of compositions in the dataset with *more than one* perovskite entry tabulated in the MP where the DFT-optimized SPuDS structure matched the MP ground state perovskite. A visual depiction of our benchmarking is shown in Fig. 5. We began with the 213 compositions discussed above, where our DFT-optimized structure matched a perovskite structure in the MP. 176 of these compositions have structures optimized from the *a*^−^*b*^+^*a*^*−*^ Glazer tilt, while the remaining 37 compositions have structures optimized from the *a*^0^*a*^0^*a*^0^ Glazer tilt. Only 39 of the 176 compositions optimized from the *a*^−^*b*^+^*a*^*−*^ Glazer tilt have *more than one* unique perovskite structure tabulated in the MP, but of these, we matched the ground state in 33 compositions (~85%). This indicates that, when present, multinary perovskite oxide structures derived from the *a*^−^*b*^+^*a*^*−*^ Glazer tilt are frequently the lowest energy perovskite tabulated in the MP. We also matched the MP ground state perovskite for 10 of the 13 compositions optimized from the *a*^0^*a*^0^*a*^0^ Glazer tilt. This suggests that octahedral tilting does not typically stabilize multinary perovskite oxide structures when SPuDS predicts no tilting.

## Usage Notes

The dataset reported in this study is the largest compilation of multinary perovskite oxides to date. This dataset can be data-mined and/or used to train machine-learning models that can elucidate structural and stability trends that will accelerate the screening of potentially interesting compositions for targeted applications. For example, the structures reported, which were optimized with GGA+U DFT, can be used as starting structures for further optimization using higher-level methods, such as SCAN or RPA. Furthermore, this dataset can be used to screen for potentially stable perovskite compositions using a *ΔH*_*d*_ cutoff energy. Importantly, this dataset can be rapidly and systematically expanded by optimizing additional SPuDS-generated ${A}_{0.5}{A}_{0.5}^{{\prime} }{B}_{0.5}{B}_{0.5}^{{\prime} }{O}_{3}$ perovskite structures using MP-compatible DFT. Additional DFT calculations that could be performed on the structures in the reported dataset, which include magnetic sampling to more accurately predict electronic properties such as band gaps and effective masses, should incur reduced computational expense because the reported bulk structures have already been optimized with DFT.

## Supplementary information

Supplementary Table 1

## Data Availability

We used the pymatgen python package, which is open-source software under the Massachusetts Institute of Technology License, for materials analysis as well as the generation of VASP inputs and CIF files. The VASP DFT code used is accessible under a paid license, copyrighted by the University of Vienna, Austria. Initial structures were generated with SPuDS DOS version >2.20.08.06 (https://www.unf.edu/~michael.lufaso/spuds/) using a custom high-throughput python wrapper available on GitHub (https://github.com/zaba1157/PySPuDS). Perovskite/non-perovskite classifications were performed using a custom python package that is available on GitHub (https://github.com/rymo1354/crystal_motifs) and based on the pymatgen and NetworkX graph network python packages.
